# Novel amino acid substitutions in Protoporphyrinogen oxidase 1 endow resistance to PPO-inhibiting herbicides in *Bassia scoparia*

**DOI:** 10.3389/fpls.2026.1904705

**Published:** 2026-09-11

**Authors:** Aimone Porri, Quincy D. Law, Charles M. Geddes, Joseph Ikley, Samuel Willingham, Philipp Johnen, Ingo Meiners, Sama Al-Sammarraie, Kim Crommar, Pieter B. F. Ouwerkerk, Michael Betz, Brian M. Jenks, Heike Heiser, Fabienne Baumann, Frank Braendle, Jens Lerchl

**Affiliations:** 1 BASF SE, Ludwigshafen am Rhein, Germany; 2 Iowa State University of Science and Technology, Ames, AI, United States; 3 Agriculture and Agri-Food Canada, Lethbridge, AB, Canada; 4 North Dakota State University, Fargo, ND, United States; 5 BASF SE Corporation, Research Triangle Park, NC, United States; 6 BASF SE, Ludwigshafen, Germany; 7 BASF SE Antwerpen NV, Antwerp, Belgium; 8 Identxx, Stuttgart, Germany

**Keywords:** *B. scoparia*, herbicide resistance, PPO, PPO-inhibiting herbicides, weeds

## Abstract

PPO-inhibiting herbicides are widely used to manage weeds in different cropping systems, yet resistance evolution threatens their long-term efficacy. Here, we investigated the molecular basis of resistance to PPO-inhibiting herbicides in *Bassia scoparia* biotypes collected from four locations in North Dakota, USA. Greenhouse dose–response assays revealed high levels of resistance to saflufenacil and carfentrazone-ethyl, while fomesafen retained full efficacy across all biotypes. Resistant plants did not show increased copy number or elevated expression of *PPX1* or *PPX2*. Sequencing of survivor plants revealed conserved PPO2 protein sequences, but consistent target-site substitutions at position F454 in PPO1, including F454I (Phenylalanine, to Isoleucine) F454L (Phenylalanine, to Leucine), and F454V (Phenylalanine, to Valine). *In vitro* enzyme assays demonstrated that these substitutions impair PPO1 enzyme sensitivity to saflufenacil and carfentrazone, but not to fomesafen. Ectopic expression of *B. scoparia PPX1* encoding F454I, L and V variants in *Arabidopsis thaliana* conferred tolerance to saflufenacil and carfentrazone-ethyl, but not to fomesafen, supporting greenhouse and *in vitro* results. Molecular modeling indicated that the conformational flexibility and interaction profile of fomesafen enables it to maintain binding to mutated PPO1 enzyme variants, in contrast to the more rigid structures of saflufenacil and carfentrazone. A yeast-based complementation system further confirmed that F454 substitutions decrease herbicide sensitivity. In addition, developmental profiling showed distinct expression patterns of *PPX1* and *PPX2* during early growth stages in *B. scoparia* and *Amaranthus palmeri*, highlighting isoform-specific roles. Together, these findings represent the first reported PPO1 target-site amino acid substitutions in a broadleaf weed species as a key mechanism of resistance and highlight that fomesafen is effective to control resistant *B. scoparia* populations.

## 1 Introduction

Herbicide resistance is a major challenge in chemical weed control, as resistant populations can develop faster than new active ingredients are discovered and commercialized (Gaines et al., 2021). Despite this challenge, reliance on herbicides for weed management continues to increase as urbanization results in dwindling rural populations relocating to urban centers and concomitant decreasing on-farm labor (Peterson et al., 2018). The resultant selection pressure for resistance caused by greater reliance on herbicides and fewer effective herbicide options has led to a steady increase in herbicide-resistant weeds worldwide (Heap, 2026). Stacking of resistance traits in multiple herbicide-resistant weed populations further complicates weed control (Torra et al., 2021), leading to a simultaneous need for new active ingredients to manage them combined with integrated weed management strategies to curb further selection and spread.

*B.scoparia* is a competitive monoecious diploid (2n = 18), warm-season (C4) summer-annual tumbleweed that is challenging to manage in croplands and ruderal areas due to its unique biology. Early emergence and prolonged emergence periodicity (Schwinghamer and Van Acker, 2008; Dille et al., 2017; Kumar et al., 2018), tolerance to abiotic stress, high fecundity (Friesen et al., 2009; Geddes and Sharpe, 2022), high genetic diversity (Martin et al., 2020; Beckie et al., 2018), and efficient pollen- and seed-mediated gene flow (Beckie et al., 2016) contribute to the persistence and spread of this broadleaf weed in arid and semi-arid environments. Recent surveys of the United States and Canada identified *B. scoparia* as the second- and third-most troublesome weed in broadleaf and grass crops, respectively (Van Wychen, 2023, 2025). *B. scoparia* was the sixth most-abundant weed present after postemergence weed control among 4,077 annual-cropped fields surveyed in the Canadian Prairies, where it has increased rapidly in the past decade (Leeson et al., 2025). Uncontrolled *B. scoparia* plants can cause severe (>90%) yield losses in crops such as corn (*Zea mays* L.), sorghum [*Sorghum bicolor* (L.) Moench ssp. bicolor], sugar beet (*Beta vulgaris* L.), and sunflower (*Helianthus annuus* L.) due to their competitiveness and resource-independent interference (Geddes and Sharpe, 2022). Indeterminate growth often results in lush green *B. scoparia* plants in senesced crops during harvest, and concomitant harvest inefficiencies.

*B. scoparia* populations in North America have been reported with resistance to five herbicide sites of action, including auxin mimics, and inhibitors of acetolactate synthase (ALS), photosystem II (PSII), 5-enolpyruvylshikimaate-3-phosphate synthase (EPSPS), and protoporphyrinogen oxidase (PPO) [Herbicide Resistance Action Committee (HRAC) Groups 4, 2, 5, 9, and 14, respectively] (Heap, 2026). Resistance to ALS- and EPSPS-inhibiting herbicides is widespread among *B. scoparia* populations in the North American Great Plains, while auxin mimic and PSII inhibitor resistance occur less frequently and vary geographically within this region (Geddes et al., 2022, 2023; Sharpe et al., 2023; Araujo et al., 2024; Dhanda et al., 2025). *B. scoparia* populations with resistance to these four sites of action have been known to occur for over a decade (Kumar et al., 2019). In contrast, PPO inhibitor resistance was confirmed only recently in *B. scoparia* populations collected after control failures in Saskatchewan, Canada in 2021, and two locations in North Dakota, USA in 2022 (Geddes et al., 2025a; Ikley et al., 2026). All three populations were highly resistant to foliar-applied saflufenacil and carfentrazone-ethyl, while other PPO-inhibiting herbicides were not tested. Screening of *B. scoparia* survey (n = 882) and grower-submitted (n = 14) samples in Western Canada identified an additional PPO inhibitor-resistant population in Alberta, Canada in 2023 (Geddes et al., 2025b).

PPO–inhibiting herbicides are used across agronomic and specialty crops to control broadleaf and some grass weeds through pre-plant burndown, preemergence residual, and postemergence applications (Yadav et al., 2020; Torbiak et al., 2021a; Torbiak et al., 2021b; Torbiak et al., 2022; Torbiak et al., 2024; Barker et al., 2023). These herbicides function by inhibiting the PPO enzyme, a key component in the chlorophyll biosynthesis pathway, leading to a rapid accumulation of phytotoxic intermediates that cause cell membrane disruption and plant death. Adoption of PPO-inhibiting herbicides has increased in recent years, largely driven by the ongoing evolution and spread of ALS and EPSPS inhibitor-resistant weeds (Dayan et al., 2018; Barker et al., 2023). Use may continue to expand, as PPO represents a versatile enzymatic target, with more than 100,000 experimental molecules identified as potential inhibitors (Barker et al., 2023). Previous research explored combining structural features from commercial PPO inhibitors as a strategy to overcome PPO inhibitor resistance (Mattison et al., 2023).

Protoporphyrinogen oxidase (PPO) catalyzes the final common step in chlorophyll and heme biosynthesis by oxidizing protoporphyrinogen IX to protoporphyrin IX within plastids (Barker et al., 2023). PPO-inhibiting herbicides block this reaction, resulting in the accumulation of photodynamic intermediates that trigger rapid lipid peroxidation, membrane disruption, and plant death (Dayan et al., 2018; Barker et al., 2023). In higher plants, PPO activity is encoded by two nuclear genes, *PPX1* and *PPX2*, which produce the PPO1 and PPO2 isoforms, respectively.

Although PPO1 and PPO2 catalyze the same biochemical reaction, they have followed distinct evolutionary trajectories with respect to herbicide resistance. To date, most confirmed target-site resistance mechanisms in weeds have been associated with mutations in *PPX2*, including the ΔG210 deletion and substitutions at residues such as R128 and G399 (Dayan et al., 2018; Barker et al., 2023; Riechers et al., 2024). These mutations have been identified in broadleaf weed species such as *Amaranthus* spp. and frequently confer resistance to multiple PPO-inhibiting herbicides. Earlier studies suggested that *PPX2* represented a more evolutionarily favorable target for resistance because PPO2 was proposed to localize to both chloroplasts and mitochondria, whereas PPO1 was considered exclusively chloroplastic (Watanabe et al., 2001; Dayan et al., 2018). However, more recent work has demonstrated that both PPO1 and PPO2 are localized in chloroplasts in several plant species, including *Arabidopsis thaliana, Spinacia oleracea, and Amaranthus* spp (Hedtke et al., 2023; Wittmann et al., 2024). These findings suggest that the predominance of PPX2-mediated resistance is unlikely to be explained solely by subcellular localization and may instead reflect differences in evolutionary constraints, structural tolerance to mutation, or gene regulation (Barker et al., 2023; Riechers et al., 2024).

Interestingly, Dayan et al. (2018) identified B. scoparia as a species possessing the same *PPX2* tandem repeat that facilitates the ΔG210 deletion in *A. tuberculatus and A. palmeri*, suggesting that this species might also evolve resistance through the well-established PPX2 pathway. However, the molecular basis of PPO-inhibitor resistance in *B. scoparia* has not yet been elucidated, and whether resistance follows the canonical *PPX2*-mediated mechanism or involves alternative evolutionary pathways remains unknown.

### 1.1 Objectives

While the presence of PPO inhibitor resistance in *B. scoparia* has been confirmed (Geddes et al., 2025a), the specific molecular and biochemical mechanisms driving it have not been elucidated. Thus, the objectives of this research were to: 1) test resistance to saflufenacil, carfentrazone-ethyl, and fomesafen in four North Dakota biotypes; 2) investigate the roles of target-site mutations, gene amplification, and gene overexpression as potential resistance mechanisms; and 3) functionally validate the impact of identified mutations on herbicide sensitivity.

## 2 Materials and methods

### 2.1 Plant material and greenhouse herbicidal treatments

A greenhouse experiment was conducted to evaluate whole-plant response to foliar applications of saflufenacil (Sharpen SC – Saflufenacil 342 ga/L – BASF US), carfentrazone-ethyl (Aim EC - carfentrazone-ethyl 240 ga/L – FMC Ag US) and fomesafen (Reflex – fomesafen 240 ga/L – Syngenta US), on four *B. scoparia* biotypes collected from field populations near Minot, Mandan, Mott and Berthold, North Dakota in 2023. Additionally, a known sensitive biotype was included for comparison.

For each biotype, the experiment was designed as a three-factor (biotype x herbicide x herbicide rate) factorial on a randomized complete block design (RCBD), with 12 replicates. Seeds from each biotype were sown in greenhouse flats measuring 25 by 50 cm, containing commercial potting mix (Fafard Germinating Mix; Sun Gro Horticulture, Agawam, MA, USA), and transplanted into 760-cm^3^ pots (Ray Leach SC-10 Super Cell Cone-tainers; Stuewe & Sons, Tangent, OR, USA) filled with the same potting mix as sown once seedlings reached 3 cm in height. Plants were watered daily and fertilized weekly with a micro- and macronutrient fertilizer (Jack’s Classic Professional 20-20-20, JR Peters Inc., Allentown PA, USA) until they reached the 7 to 10 cm in height, at which time herbicide applications were made using a track-mounted research sprayer (Generation III Research Sprayer, DeVries Manufacturing, Hollandale, MN) calibrated to deliver 140 L ha^-1^ at 207 kPa via an Turbo TeeJet^®^ TT110015 nozzle (TeeJet Technologies, Glendale Heights, IL). Herbicide treatments included seven rates of carfentrazone-ethyl (8.77 to 210 g ai ha^-1^, projected labeled 1x use rate = 17.5 g ai ha^-1^), saflufenacil (12.5 to 300 g ai ha^-1^, labeled 1x use rate = 25 g ai ha^-1^), or fomesafen (105 to 2530 g ai ha^-1^, labeled 1x use rate = 210 g ai ha^-1^) with methylated seed oil (MSO Ultra, Precision Laboratories, Waukegan, IL) added to each herbicide treatment at 1% v v^-1^. Treatments were applied at 0.5x, 1x, 2x, 4x, 6x, 8x and 12x of the recommended field use rate. Greenhouse environmental conditions included a 14:10 h light:dark photoperiod, where natural light was supplemented with high-pressure sodium bulbs delivering 1100 μmol m^-2^ s^-1^ photon flux during daylight hours, and day/night temperatures of 30 and 25 °C, respectively. Visual estimates of *B. scoparia* control were recorded at 7, 14 and 21 days after application (DAA) utilizing a 0 to 100 scale, where 0 = no injury and 100 = complete plant death. Data was subjected to ANOVA statistical analysis.

### 2.2 Yeast assay

A haploid strain YKC027 (MATα his3Δ1 leu2Δ0 ura3Δ0 lys2Δ0 hem14::kanMX4) knock-out for the single *PPX* gene *HEM14* (Glerum et al., 1996; Camadro and Labbe, 1996) was isolated by tetrad dissection and mass-sporulation of strain YER014 BY4743 which is heterozygous for HEM14/hem14::kanMX4 and which originates from the yeast genome deletion collection (Transomic, AL, USA). Haploids were selected on G418 containing medium thereby selecting for the *hem14::kanMX* knock-out locus which were genotyped with the ABCD primer system (Cherry et al., 2011; Giaever and Nislow, 2014). The hem14::kanMX haploid strain YKC027 was further genotyped and phenotyped by selection on minimal SC plates to establish phenotypes for lysine and methionine auxotrophy versus prototrophy. Constructs *p416ADH-AmPPX1* and *p416ADH-AmPPX2* were based on *A. palmeri PPX*1 and *PPX2* sequences, respectively. The mating type of YKC027 was confirmed with colony PCR using the primers described by Huxley et al. (1990). Although YKC027 lacks mitochondrial respiration because no active *PPX* gene is present, it will still grow on standard YPD medium containing glucose as fermentable carbon source. If glucose is replaced by 2% ethanol and 3% glycerol as non-fermentable carbon sources in Yeast Peptone Glycerol Ethanol (YPGE) medium, YKC027 cannot grow anymore since yeast needs mitochondria with a fully functional and heme-dependent respiration system to be able to grow on glycerol and ethanol. Growth on YPGE can be restored again by transforming YKC027 with a plant *PPX* gene construct. With the conditional lethality screening system that we created in this way, we were able to use a simple plate-based screening system to check for herbicide sensitivity versus tolerance conferred by plant *PPX* gene variants since inhibition of PPO activity by an herbicide will inhibit growth when cultured on YPGE. Plant *PPX* constructs for complementation of the *hem14::kanMX4* locus in YKC027 were synthetically made (Azenta-Genewiz, Germany) in the URA3 ARS-CEN vector *p416ADH* in which the gene of interest is driven by the *ADH1* promoter (Mumberg et al., 1995), transformed into yeast by a LiAc transformation method (Gietz and Schiestl, 2007) and selected for uracil prototrophy on SC (Synthetic Complete) 2% glucose plates respecting the genotype from **Table 1**. Apart from the wild-type *PPX1* sequence from *B. scoparia* (represented by construct p416ADH-BsPP01 in yeast strain YSA024), three different constructs were tested (**Supplementary Table 3**, Yeast strains YSA025 to YSA027 harboring constructs p416ADH-BsPPO1-F454I, p416ADH-BsPPO1-F454V, p416ADH-BsPPO1-F454L). For herbicide screens, colonies were grown on YPGE plates with or without PPO-inhibiting herbicides to check for tolerance levels. Saflufenacil, carfentrazone, and fomesafen were prepared as 100 mM stock solutions in 100% DMSO. For the yeast growth assays, these stocks were further diluted to 10 mM or 1 mM in 100% DMSO and subsequently added to the medium as 1000-fold stock solutions. For assays using 100 µM herbicide, the final DMSO concentration in the medium was 0.1%. Saflufenacil was tested at a final concentration of 500 µM, resulting in a final DMSO concentration of 0.5%. Fomesafen was applied directly onto the surface of the solidified YPGE medium to a final concentration of 500 µM from a 100 mM stock solution. These DMSO concentrations are well below those reported to inhibit yeast growth (Sadowska-Bartosz et al., 2013).

**Table 1:** Amino acid residues at key PPO2 target-site positions in *B. scoparia* plants surviving treatment with PPO-inhibiting herbicides.

A							
Population	Survivor ID	*B. scoparia* PPO2	R94	G176	L327	G349	G365
		*Amaranthus* PPO2	R128	G210	V361	G383	G399
Berthold	Survivor 1		♢ R	♢ G	♢ L	♢ G	♢ G
	Survivor 2		♢ R	♢ G	♢ L	♢ G	♢ G
	Survivor 3		♢ R	♢ G	♢ L	♢ G	♢ G
Mandan	Survivor 1		♢ R	♢ G	♢ L	♢ G	♢ G
	Survivor 2		♢ R	♢ G	♢ L	♢ G	♢ G
	Survivor 3		♢ R	♢ G	♢ L	♢ G	♢ G
	Survivor 4		♢ R	♢ G	♢ L	♢ G	♢ G
Minot	Survivor 1		♢ R	♢ G	♢ L	♢ G	♢ G
	Survivor 2		♢ R	♢ G	♢ L	♢ G	♢ G
	Survivor 3		♢ R	♢ G	♢ L	♢ G	♢ G
Mott	Survivor 1		♢ R	♢ G	♢ L	♢ G	♢ G
	Survivor 2		♢ R	♢ G	♢ L	♢ G	♢ G
	Survivor 3		♢ R	♢ G	♢ L	♢ G	♢ G
B							
Population	Survivor ID	*B. scoparia* PPO2	R94	G176	L327	G349	G365
		*Amaranthus* PPO2	R128	G210	V361	G383	G399
Berthold	Survivor 1		♢ R	♢ G	♢ L	♢ G	♢ G
	Survivor 2		♢ R	♢ G	♢ L	♢ G	♢ G
	Survivor 3		♢ R	♢ G	♢ L	♢ G	♢ G
Mandan	Survivor 1		♢ R	♢ G	♢ L	♢ G	♢ G
	Survivor 2		♢ R	♢ G	♢ L	♢ G	♢ G
	Survivor 3		♢ R	♢ G	♢ L	♢ G	♢ G
	Survivor 4		♢ R	♢ G	♢ L	♢ G	♢ G
Minot	Survivor 1		♢ R	♢ G	♢ L	♢ G	♢ G
	Survivor 2		♢ R	♢ G	♢ L	♢ G	♢ G
	Survivor 3		♢ R	♢ G	♢ L	♢ G	♢ G
Mott	Survivor 1		♢ R	♢ G	♢ L	♢ G	♢ G
	Survivor 2		♢ R	♢ G	♢ L	♢ G	♢ G

Individual survivors from four field-collected populations (Berthold, Mandan, Minot, and Mott) were sampled after surviving labeled field rates (1x) of either (A) saflufenacil or (B) carfentrazone-ethyl. Sanger sequencing was used to identify amino acid residues at known resistance-associated positions in the *B. scoparia* PPO2 protein (R94, G176, L327, G349, G365) and their orthologous positions in *Amaranthus* PPO2 (R128, G210, V361, G383, G399).

The integrity of the *PPX* complementation constructs in yeast was checked by colony PCR and sequencing (LGC Genomics or Plasmidsaurus) using primers ProADH Fw1 gctatcaagtataaatagacctg and Tcyc1 Rev1 gtataatgttacatgcgtacaC.

### 2.3 Copy number variation and expression analysis of *B. scoparia PPO1* and *PPO2*

TaqMan™ technology was used to determine gene copy number variation (CNV) and expression of the *PPX1* and *PPX2* genes in *B. scoparia* plants that survived 1x saflufenacil and 1x carfentrazone-ethyl compared with untreated susceptible plants. The TaqMan™ assays were designed to facilitate a multiplexed approach for both target and reference genes.

Digital PCR (dPCR) was performed in a final volume of 12 µL using 1,48 µL of DNA or cDNA, 0,48 µl (0.2µM) of specific primers and 0,24 µL (0.2µM) of probe (**Supplementary Table S2**) (biomers.net GmbH, Ulm, Germany), 3 µl of QIAcuity HighMultiplex ProbePCR Kit (Qiagen, Hilden, Germany) and 5,36 µL PCR-Grade H_2_O for the triplex dPCR. Digital PCR was performed in a dPCR thermal cycler (QIAcuity One, 5plex Device, Qiagen, Hilden Germany) in a 96-well Nanoplate with 8.500 Nanowells for each sample (QIAcuity Nanoplate 8.5k 96-well) under the following conditions: 2 min at 95 °C and 55 cycles of 15 s denaturation at 95 °C; 55 s annealing and elongation at 60 °C. The following conditions were met during the imaging of the partitions. The FAM and HEX experiments were conducted with an exposure time of 500 milliseconds, with the gain set to 6. The ROX experiment utilized an exposure time of 400 milliseconds, also with the gain set to 6. The assessment of CNV and gene expression was conducted by employing Qiagen’s QIAcuity Software Suite, version 3.1.0.0. This process entailed the utilization of positive, negative, and no-template control wells (NTC), along with the determination of sample thresholds. Primers sequences are in **Supplementary Table 2**. *Actin* from *B. scoparia was used* as the reference gene for dPCR calibration.

### 2.4 Identification of target-site mutations in *B. scoparia PPX1* via cDNA sequencing

To identify target-site mutations in the *B. scoparia PPX1* gene, total RNA was extracted from young leaf tissues of plants that survived 1x saflufenacil and 1x carfentrazone-ethyl and compared with susceptible plants. Approximately 100 mg of fresh tissue was flash-frozen in liquid nitrogen and ground to a fine powder using a mortar and pestle. RNA was isolated using the RNeasy Plant Mini Kit (Qiagen, Hilden, Germany) following the manufacturer’s protocol, including an on-column DNase I treatment (Qiagen) to eliminate genomic DNA contamination. RNA integrity was assessed via 1% agarose gel electrophoresis and concentration was quantified using a NanoDrop spectrophotometer (Thermo Fisher Scientific). First-strand cDNA synthesis was performed using 1 µg of total RNA, oligo(dT) primers, and the RevertAid First Strand cDNA Synthesis Kit (Thermo Fisher Scientific) according to the manufacturer’s instructions.

The *PPX1* fragment containing the whole coding sequence was amplified from cDNA using gene-specific primers designed to span the full open reading frame of *B. scoparia*. Each 25-µL PCR reaction contained 2 µL of cDNA, 0.4 µM of each primer, 200 µM dNTPs, 1x PCR buffer, 1.5 mM MgCl₂, and 1 U Taq DNA polymerase (Thermo Fisher Scientific). PCR cycling conditions were: 95 °C for 3 min; 35 cycles of 95 °C for 30 s, 58 °C for 30 s, and 72 °C for 1 min; followed by a final extension at 72 °C for 7 min. Amplicons were confirmed on 1.2% agarose gels stained with SYBR Safe DNA Gel Stain (Invitrogen), and purified using the QIAquick PCR Purification Kit (Qiagen). Sanger sequencing was performed bidirectionally using the amplification primers. Chromatograms were analyzed using Geneious Prime, and sequences were aligned to the *B. scoparia PPX1* WT reference coding sequence. Amino acid changes resulting from nucleotide substitutions were therefore identified.

### 2.5 *B. scoparia PPX1* and *PPX2* mRNA abundance during early development

The plant material of four different growth stages (germination, 2.5 cm, 6.3 cm, and 10 cm) of *B. scoparia* and *A. palmeri* was homogenized using a mortar and pestle. The samples consisted of 10 plants per growth stage and were ground on dry ice. To ensure shelf life, the ground samples were stored at -80 °C in Falcon tubes until RNA was extracted.

RNA was extracted from the samples using the RNeasy Plant Mini Kit according to the manufacturer’s instructions. The concentration and purity of RNA were measured using a NanoDrop spectrophotometer. For cDNA synthesis, the amount of RNA extraction corresponding to 1 µg was first determined, so that 1 µg of RNA was converted to cDNA. The synthesis was performed according to the instructions of the QuantiNova Reverse Transcription Kit 200. The synthesized cDNA was stored at -20 °C until further use.

Digital PCR was performed using the dPCR system from Qiagen. Each sample was pipetted into a 96-well plate in a volume of 11.4 µL. The reaction mix was pipetted according to a template. This corresponded to a volume for 30 preparations, as 24 samples were to be measured. The reaction mixture comprised 342 µL of dPCR master mix, which contained 40 µM of specific primers, 20 µM of probes, 150.9 µL of ddH₂O and 97.5 µL of Qiagen PCR Kit reagent. The master mix was then mixed with 1.6 µL cDNA (1:5 diluted) sample and pipetted into the wells.

Specific fluorescent markers were used to ensure reliable detection of the *PPX1* and *PPX2* mRNA expression. In *B. scoparia*, *PPX1* expression was detected using fluorochrome FAM, while *PPX2* was labeled HEX. In the case of *A. palmeri*, *PPX*1 mRNA abundance was detected by the fluorescent dye ROX and *PPX2* by FAM. This specific labeling enabled a clear differentiation of the target genes in the respective species. Amplification was performed under the following conditions: Initial denaturation at 95 °C for 2 min, followed by 40 cycles at 95 °C for 10 s and 60 °C for 40 s. Primers sequences are in **Supplementary Table S2**.

### 2.6 Recombinant expression, purification, and *in vitro* inhibition assays of *B. scoparia* PPO1 wildtype and mutant enzymes

To investigate the functional effects of amino acid substitutions at position F454 in *B. scoparia* PPO1, three variants F454I, F454L, and F454V of the PPO1 enzyme were generated and evaluated for enzymatic activity and herbicide sensitivity. The full-length *B. scoparia PPX1* coding sequence was synthesized *de novo* and cloned into the pRSetB expression vector (Invitrogen, Carlsbad, CA, USA) using *BamH I* and *Hind III* restriction sites. A N-terminal hexahistidine tag was included to facilitate purification. Recombinant constructs were transformed into *Escherichia coli* strain BL21(DE3) pLysS (Novagen, EMD Millipore, Billerica, MA, USA), and transformants were selected on LB agar containing 100 µg mL^-1^ ampicillin and 34 µg mL^-1^ chloramphenicol.

For protein expression, a single colony was used to inoculate 3 mL LB medium with antibiotics and incubated at 37 °C with shaking (200 rpm) for 6 h. A 20-µL aliquot was transferred into 20 mL fresh LB medium and incubated overnight. The next day, 100 µL of the overnight culture was inoculated into 100 mL ZYM-5052 autoinduction medium (see Supplemental Data for preparation), supplemented with antibiotics, and incubated at 37 °C for 5 h, followed by 21 h at 25 °C.

Cells were harvested by centrifugation at 6,000 × g for 30 min at 4 °C. Cell pellets were resuspended in lysis buffer [10 mL g^-1^ pellet; 50 mM NaH₂PO₄, 100 mM NaCl, 5 mM imidazole, 5% (v v^-1^) glycerol, pH 7.5, 20 mg mL^-1^ lysozyme, 30 U mL^-1^ DNase I] supplemented with protease inhibitors (complete EDTA-free; Roche Diagnostics, Mannheim, Germany). Suspensions were sonicated on ice (3 min, 90% amplitude, 30-s intervals). Cell debris was removed by centrifugation at 38,000 × g for 30 min at 4 °C, and 2 mL of 5 M NaCl was added to the clarified supernatant.

For purification, a 500-µL bed volume of HisPur Ni-NTA resin (Thermo Fisher Scientific, IL, USA) was equilibrated with buffer (20 mM NaH₂PO₄, 50 mM NaCl, 5 mM imidazole, 5 mM MgCl₂, 17% glycerol, 0.1 mM EDTA, pH 8.0). The supernatant was passed through the resin, washed with 5.6 mL wash buffer (20 mM NaH₂PO₄, 50 mM NaCl, 5 mM imidazole, 17% glycerol, pH 7.5), and the bound protein was eluted with 1 mL elution buffer (20 mM NaH₂PO₄, 50 mM NaCl, 250 mM imidazole, 17% glycerol, pH 7.5). Protein concentrations were determined using a Scandrop nanovolume spectrophotometer (Analytikjena, Life Science, Germany). Purity and solubility were assessed by SDS-PAGE (10%) using 2.5 µg protein per lane.

PPO1 activity was assayed using a fluorescence-based method (excitation: 405 nm; emission: 630 nm) in a 187-µL reaction containing 100 mM Tris–HCl, 1 mM EDTA, 5 mM DTT, 0.0085% Tween 80, and 15 µL enzyme in resuspension buffer (50 mM Tris–HCl, pH 7.3, 3.2 mM EDTA, 20% v/v glycerol). Enzyme concentration varied by mutant to equalize maximum fluorescence in the absence of inhibitor and under saturating substrate conditions. Fluorescence units per min were calculated and normalized to WT PPO1 activity.

All PPO1 variants were subjected to dose-response assays. PPO inhibitors (Pestanal standards, Sigma-Aldrich, Steinheim, Germany) were dissolved in 80% DMSO. Ten concentrations (10 µL; range: 5 × 10^-5^ to 5.12 × 10^-1^² M) were added to the reaction mixture and incubated for 30 min at room temperature. Protoporphyrinogen IX (3.24 µM; prepared from protoporphyrin IX via sodium amalgam reduction was then added. Fluorescence was recorded for 30.25 min (33 cycles of 55 s) using a microplate reader (CLARIOstar, BMG LabTech, Germany). Percent inhibition was calculated relative to untreated (positive) and no-enzyme (negative) controls. Assays were conducted in duplicate. IC_50_ values (concentration of inhibitor reducing PPO1 activity by 50%) were estimated by nonlinear regression (three- or four-parameter log-logistic models). Resistance factors were calculated by dividing the IC_50_ of each mutant variant by that of WT *B. scoparia* PPO1.

## 3 Results

### 3.1 Confirmation of PPO inhibitor resistance in *B. scoparia* biotypes collected from different North Dakota locations

**Figure 1:**
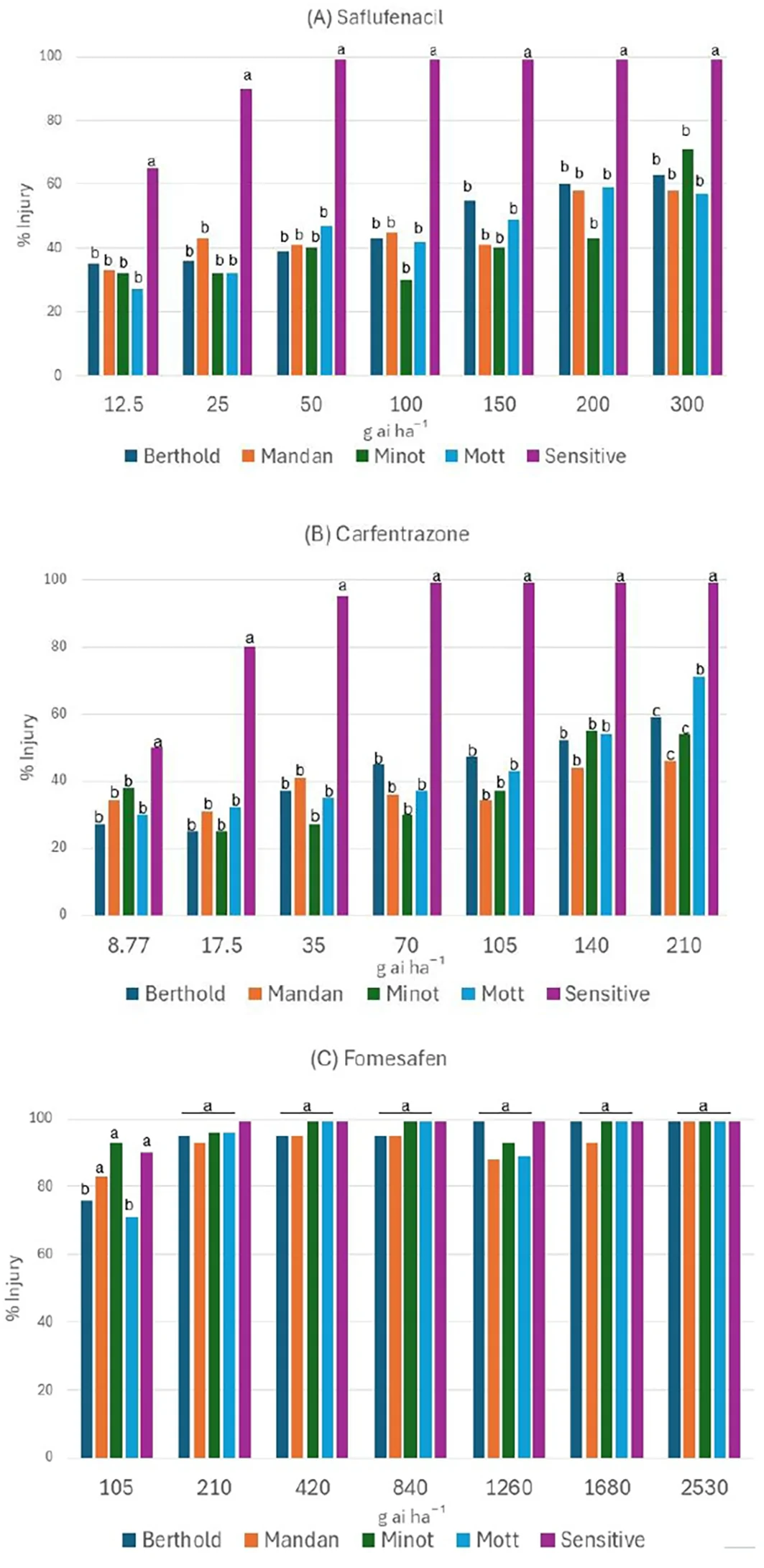
Dose–response of *B. scoparia* populations to three PPO-inhibiting herbicides under greenhouse conditions. **(A)** saflufenacil, **(B)** carfentrazone, and **(C)** fomesafen were applied at increasing rates (x-axis, g ai ha^-1^) to four field-collected populations (Berthold, Mandan, Minot, and Mott) and a sensitive reference population was used as control. The lowest herbicide rate (leftmost value on the x-axis) corresponds to 0.5x the labeled field use rate. Visual injury was assessed 14 DDA. Different letters indicate statistically significant differences among populations at each herbicide rate, as determined by one-way ANOVA followed by Tukey’s honestly significant difference (HSD) test (α = 0.05).

A clear dose-dependent injury pattern was observed following saflufenacil application. The sensitive check exhibited near-complete visual injury (~100) at all application rates above 12.5 g ai ha^-1^ (0.5×), confirming the herbicide’s effectiveness in susceptible backgrounds (**Figure 1A**). At the lowest dose 12.5 g ha^-1^ (0.5x) of saflufenacil, Berthold, Mandan, Minot, and Mott biotypes exhibited moderate injury levels below 40% while the sensitive biotype showed more than 60% injury. As expected, as the dose increased to 300 g ha^-1^ (12x), injury progressively intensified in Berthold, Mandan, Minot, and Mott reaching approximately 60–75% for all biotypes while the sensitive plants were fully controlled, indicating lack of full herbicidal control in the four *B. scoparia* biotypes even at very high rates. At 25 g ha^-1^ (1x) the four biotypes showed 40% injury or less, while the sensitive plants reached 90% injury, indicating herbicidal loss of efficacy at the recommended field dose rate of saflufenacil. Thus, *the B. scoparia* biotypes tested were highly resistant to saflufenacil.

Responses to carfentrazone-ethyl were similar to those observed for saflufenacil. The sensitive check once again showed severe injury (~100%) across most rates, confirming herbicidal activity (**Figure 1B**). At the lowest dose of 8.77 g ha^-1^, injury among the biotypes ranged between 25–40%. With increasing doses, injury levels rose in all biotypes and at the recommended field dose rate of 17.5 g ha^-1^ (1x) all four *B. scoparia* biotypes showed injury less than 40%, while the sensitive plants reached 80% injury, demonstrating significant resistance to carfentrazone-ethyl.

Fomesafen treatments led to high levels of visual injury across all biotypes, with little differentiation among them (**Figure 1C**). Even at the lowest dose of 105 g ha^-1^ (0.5x), injury levels were significant for all tested biotypes, closely matching the response of the sensitive check. As the dose increased to 2,530 g ha^-1^, the injury remained uniformly high, with all biotypes approaching full damage (~95–100%). At the 210 g ha^-1^ (1x) field rate, all biotypes and the sensitive standard were near-fully controlled with no statistical difference among them. This uniformity in response suggests a general susceptibility to fomesafen in the tested biotypes, with minimal variability. These data together strongly indicate that the tested *B. scoparia* biotypes were highly resistant to carfentrazone-ethyl and saflufenacil, while fomesafen retained high herbicidal efficacy.

### 3.2 Copy number and expression of *PPX1* and *PPX2* remains unchanged in resistant *B. scoparia* plants

**Figure 2:**
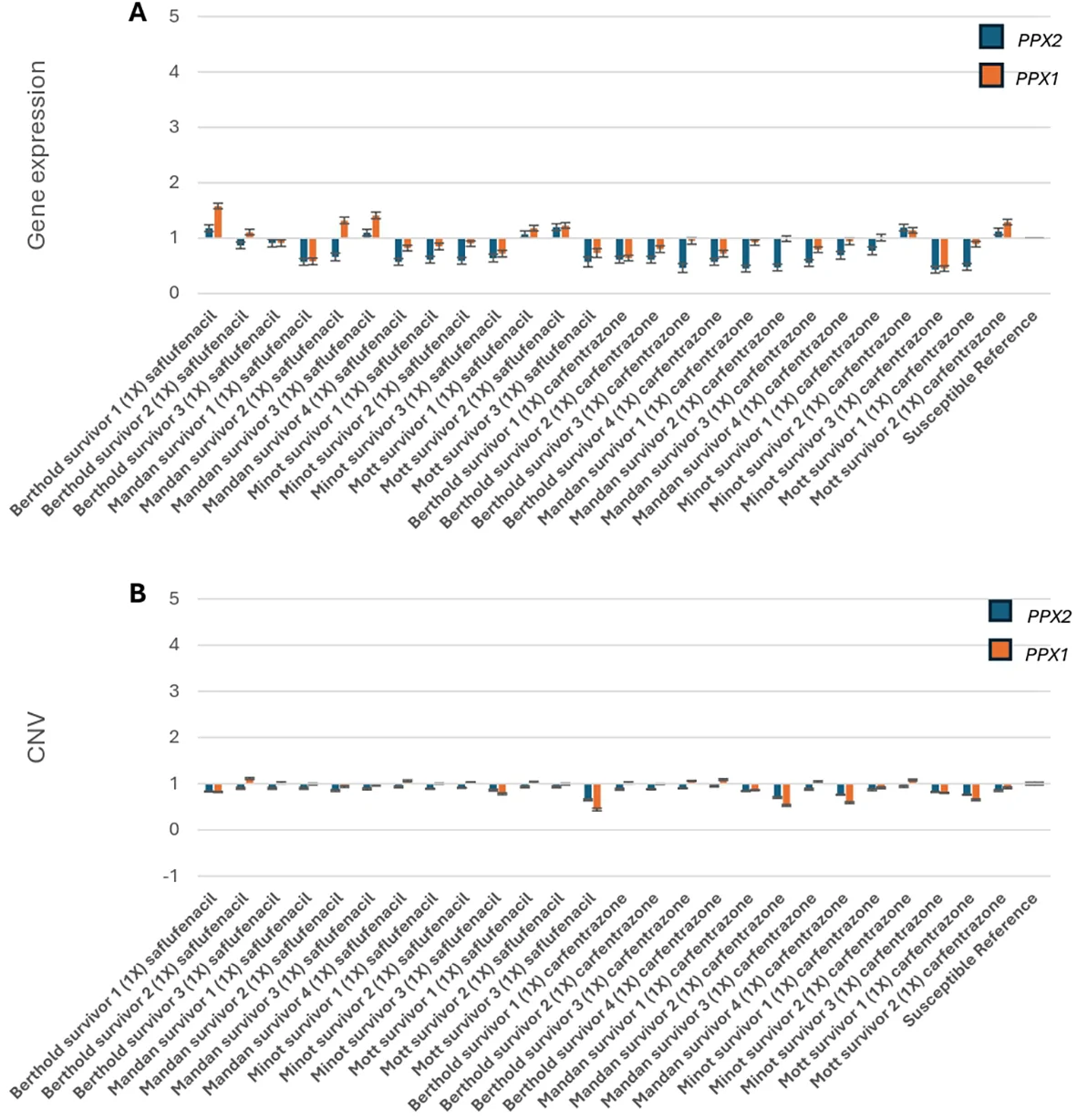
Copy number variation (CNV) and gene expression of *PPX1* and *PPX2* in *B. scoparia* individuals surviving treatment with PPO-inhibiting herbicides. **(A)** CNV was assessed by digital qPCR using actin as a reference gene, and values were normalized to a sensitive reference population (set to 1.0). **(B)** Gene expression was measured by digital qPCR in the same individuals, normalized to *actin* and relative to the sensitive population. Plants were collected following survival to labeled field rates (1x) of either saflufenacil or carfentrazone. Bars represent 95% confidence intervals (CI95).

To assess the potential involvement of gene amplification in PPO inhibitor resistance, CNV of *PPX1* and *PPX2* was evaluated in *B. scoparia* plants that survived 1X treatment of saflufenacil and carfentrazone-ethyl relative to a sensitive control. As shown in **Figure 2A**, no consistent increase in copy number was observed for both genes across resistant individuals from the four biotypes. Fold-change values clustered around or below unity, indicating that *PPX1* and *PPX2* were not genomically amplified in the resistant plants. Thus, CNV is unlikely to be a major mechanism contributing to PPO inhibitor resistance in these *B. scoparia* plants. In addition, quantification of *PPX1* and *PPX2* transcript levels across *B. scoparia* survivor plants revealed limited variation in gene expression with most samples showing mRNA abundance levels comparable to the susceptible reference, with only minor fluctuations observed between treatments or locations (**Figure 2B**). Together, these results suggest that *PPX1* and *PPX2* expression remain stable in *B. scoparia* survivor plants indicating that neither CNV nor expression changes in *PPX1* and *PPX2* are likely to mediate resistance to saflufenacil and carfentrazone-ethyl.

### 3.3 PPO1 F454 mutations are associated with resistance in *B. scoparia* survivor plants

To elucidate the molecular basis of resistance, *PPX1* and *PPX2* cDNAs were sequenced in survivor plants following treatment with 1x rates of saflufenacil or carfentrazone-ethyl. Sanger sequencing was employed to identify possible amino acid substitutions derived from nucleotide changes.

Sequencing of the *PPX2* cDNA revealed complete conservation of amino acid residues at all known target-site positions (R94, G176, L327, G349, and G365 in *B. scoparia* PPO2; R128, G210, V361, G383, and G399 in *Amaranthus* PPO2 homolog). As shown in **Tables 1A, B**, all survivor plants retained WT residues across these positions, regardless of herbicide treatment or biotype origin. This absence of non-synonymous substitutions indicates that PPO2 variations in these key target site positions did not occur in the survivor plants. Substitutions at PPO2 positions I201 and V331 were detected in *B. scoparia* survivor plants treated with 1x saflufenacil **Supplementary Table 1A** or carfentrazone-ethyl **Supplementary Table 1B**. While I201 remained WT in most individuals, I/L heterozygosity was observed in two Mandan and one Berthold survivors, and a homozygous L201 variant was exclusive to two Mott survivors. At V331, most plants retained the WT valine. However, V/F heterozygotes were found in one Minot and all Mott survivors. The inconsistent occurrence of such amino acid substitutions indicates that they were unlikely to contribute to the resistant phenotype of the survivor plants. No further amino acid substitutions were detected in PPO2.

In contrast, sanger sequence analysis of the *PPX1* cDNA revealed a diverse array of amino acid substitutions consistently at position F454. As presented in **Tables 2A, B**, all survivor plants from both treatment groups exhibited either homozygous or heterozygous substitutions, including F/I, F/L, I/L, I, L, and V substitutions. The F454I substitution emerged as the most prevalent across populations, particularly in individuals from Berthold and Mandan, where it was frequently detected in a heterozygous (F/I) or homozygous (I) state; especially all three Berthold survivors and all four Mandan individuals surviving carfentrazone-ethyl.

**Table 2:** Amino acid residues at the F454 position of PPO1 in *B. scoparia* survivors to PPO-inhibiting herbicide treatment.

A		
Population	Survivor ID	*B. scoparia* PPO1 F454
Berthold	Survivor 1	♢ I
	Survivor 2	♢ F/I
	Survivor 3	♢ I
Mandan	Survivor 1	♢ I
	Survivor 2	♢ I
	Survivor 3	♢ F/L
	Survivor 4	♢ I/L
Minot	Survivor 1	♢ L
	Survivor 2	♢ V
	Survivor 3	♢ L
Mott	Survivor 1	♢ F/I
	Survivor 2	♢ F/I
	Survivor 3	♢ I
B		
Population	Survivor ID	*B. scoparia* PPO1 F454
Berthold	Survivor 1	♢ F/I
	Survivor 2	♢ I
	Survivor 3	♢ F/I
Mandan	Survivor 1	♢ F/I
	Survivor 2	♢ F/I
	Survivor 3	♢ F/I
	Survivor 4	♢ I
Minot	Survivor 1	♢ I
	Survivor 2	♢ V
	Survivor 3	♢ V
Mott	Survivor 1	♢ F
	Survivor 2	♢ F/I

Individual plants from four field-collected populations (Berthold, Mandan, Minot, and Mott) were sampled after surviving 1x labeled field rates of either (A) saflufenacil or (B) carfentrazone-ethyl. Sanger sequencing was used to identify amino acid substitutions at position F454 of the *B. scoparia* PPO1 protein. Residues are shown for each survivor. Where two amino acids are listed (e.g., F/I), this indicates heterozygosity at the locus, with one allele carrying the wild-type residue and the other the substitution.

Evidence of allelic diversity was observed not only among populations but also within them. For instance, Minot survivors exposed to saflufenacil displayed genotypic heterogeneity at F454, including individuals carrying V, L substitutions. The presence of multiple amino acid variants at a single residue suggests a high degree of selective pressure on this locus and points to the evolution of distinct mutational pathways associated with resistance. No other amino acid substitutions were detected in the PPO1 isoform.

Taken together, these results suggest that substitutions at position F454 in PPO1, F454I, F454L, and F454V are associated with the resistance phenotype in the four *B. scoparia* biotypes. In contrast, no evidence supports a role for PPO2 in herbicide resistance in these populations. The observed prevalence, allelic diversity, and zygosity of F454 substitutions across survivors from both saflufenacil and carfentrazone-ethyl further support the critical involvement of these substitutions in conferring resistance.

### 3.4 F454 substitutions reduce PPO1 sensitivity to saflufenacil and carfentrazone but not fomesafen *in vitro*

To elucidate the impact of amino acid substitutions at position F454 on the sensitivity of *B. scoparia* PPO1 to PPO-inhibiting herbicides, we evaluated the inhibitory responses of recombinant PPO1 WT and mutant enzymes (F454V, F454L, and F454I) to saflufenacil, carfentrazone, and fomesafen. IC_50_ values were determined as a measure of half-maximal inhibition, and percent inhibition was assessed at a saturating herbicide concentration (10^-4^ M) (**Table 3**).

**Table 3:** IC_50_ values of *B. scoparia* PPO1 (WT), F454V, L and I enzymes.

	IC_50_ [M]	% Inhibition at 10^-4^M	RF
Saflufenacil			
KSHCS PPO1 WT	8.60E-07	90	–
KSHCS PPO1 F454V	1.97E-05	73	23
KSHCS PPO1 F454L	4.61E-06	82	5
KSHCS PPO1 F454I	4.96E-06	80	6
Carfentrazone			
KSHCS PPO1 WT	5.20E-07	88	–
KSHCS PPO1 F454V	2.88E-05	53	55
KSHCS PPO1 F454L	1.17E-05	55	23
KSHCS PPO1 F454I	1.34E-05	52	26
Fomesafen			
KSHCS PPO1 WT	8.06E-08	98	–
KSHCS PPO1 F454V	3.49E-08	95	0.4
KSHCS PPO1 F454L	1.22E-08	98	0.2
KSHCS PPO1 F454I	3.67E-08	99	0.5

IC_50_ is expressed in molar concentrations which represents the amount of a given herbicide that inhibits 50% of the recombinant protein activity *in vitro.* % Inhibition at 10^-4^M represents the total enzyme inhibition with the highest herbicide concentration used in the assay. Resistance factor (RF) was calculated as IC_50_(mutant)/IC_50_(WT).

For saflufenacil, the WT enzyme displayed an IC_50_ of 8.60 × 10^-7^ M, with 90% inhibition at 10^-4^ M. Substitution with valine at position F454 (F454V) markedly reduced herbicide sensitivity, increasing the IC_50_ to 1.97 × 10^-5^ M and yielding a resistance factor (RF) of 23. In contrast, the F454L and F454I variants exhibited moderate resistance, with IC_50_ values of 4.61 × 10^-6^ M and 4.96 × 10^-6^ M, corresponding to RFs of 5 and 6, respectively. At 10^-4^ M of saflufenacil both mutants showed inhibition ranging 80–82%, indicating lack of full inhibition under high herbicide concentrations. Resistance was even more pronounced in response to carfentrazone. The WT enzyme showed an IC_50_ of 5.20 × 10^-7^ M, with 88% inhibition at 10^-4^ M. However, the F454V variant exhibited a 55-fold increase in resistance (IC_50_ = 2.88 × 10^-5^ M), while F454L and F454I mutants showed IC_50_ values of 1.17 × 10^-5^ M and 1.34 × 10^-5^ M, respectively, with corresponding RFs of 23 and 26. Consistently, inhibition at 10^-4^ M was substantially diminished across all three mutants (55–26%), underscoring a substantial loss of carfentrazone- efficacy in the presence of F454 substitutions. By contrast, fomesafen retained high inhibitory potency across all enzyme variants. The WT PPO1 enzyme exhibited an IC_50_ of 8.06 × 10^-8^ M and 98% inhibition at 10^-4^ M. Notably, the F454V, F454L, and F454I substitutions had minimal impact, with IC_50_ values ranging from 1.22 × 10^-8^ M to 3.67 × 10^-8^ M and RFs between 0.2 and 0.5. Inhibition at 10^-4^ M remained consistently high (95–99%) across all enzyme variants, indicating that fomesafen efficacy is largely unaffected by these target-site substitutions.

The above experiments suggest herbicide-specific effects of F454 substitutions on PPO1 inhibition. While F454 substitutions substantially impair enzyme sensitivity to saflufenacil and carfentrazone, they exert negligible influence on fomesafen activity, highlighting distinct structural interactions between PPO1 substitutions and these herbicidal chemistries.

### 3.5 Ectopic expression of F454L, I and V in *Arabidopsis* transgenic lines

**Figure 3:**
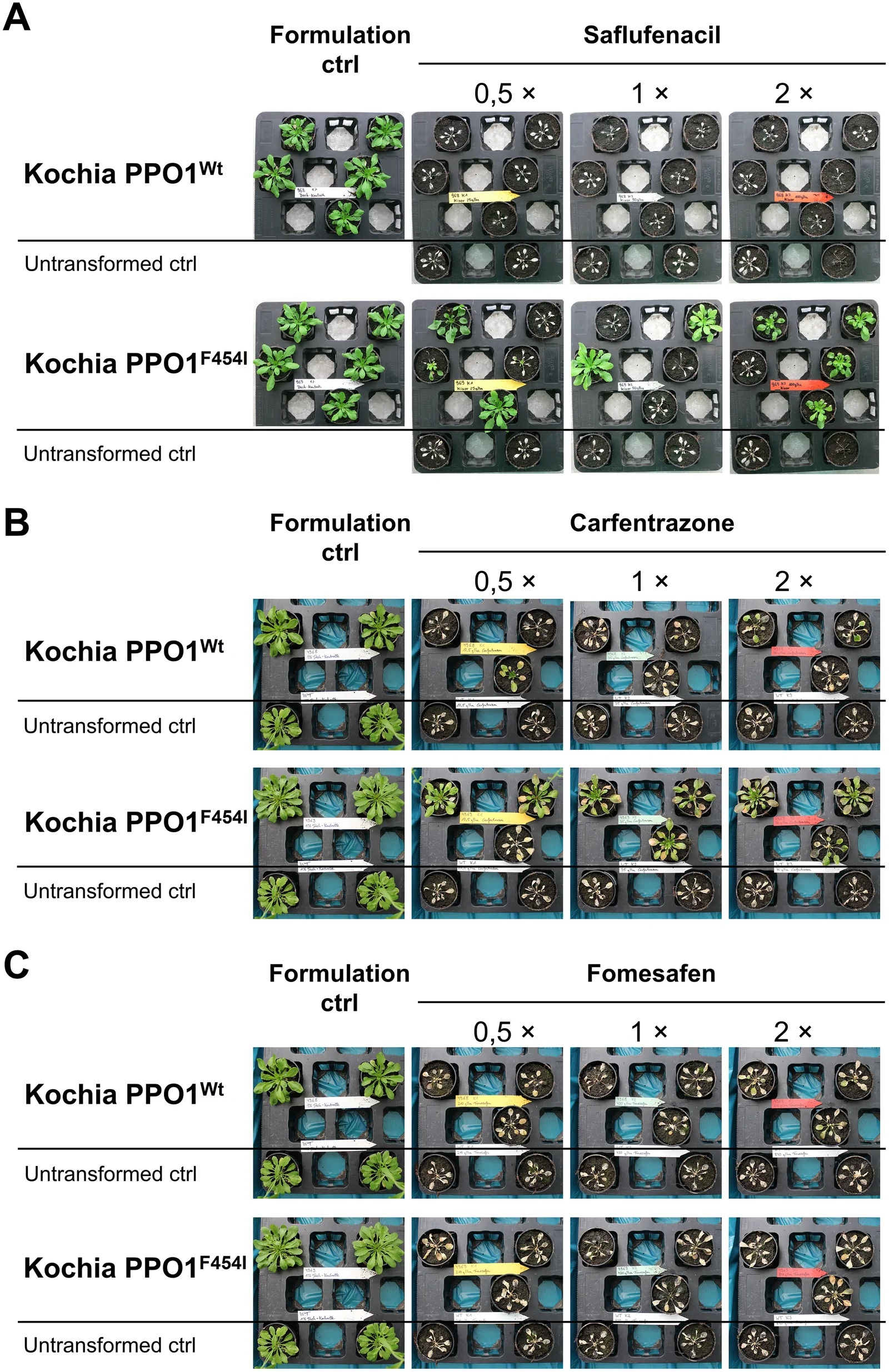
*Arabidopsis* lines in the T1 Generation ectopically expressing indicated *B. scoparia PPX* variants were sprayed with saflufenacil, carfentrazone or fomesafen. The upper three to five pots contain independent transgenic events (T1 plants, were selected with imazamox to confirm the presence of resistance gene T-DNA via AHAS herbicide tolerance) the two plants in the lowest row represent the untransformed MC24 wild type control. **(A)** Images are shown of *Arabidopsis* lines ectopically expressing *B. scoparia PPX1^Wt^*, *B. scoparia PPX1^F454I^* (upper three rows of trays) or the untransformed control (lowest row of trays) after spray treatment with the formulation control or saflufenacil (0.5x: 12.5 g a.i./ha, 1x: 25 a.i./ha or 2x: 50 a.i./ha field use rates) 11 DDA. **(B)** Images are shown of *Arabidopsis* lines ectopically expressing *B. scoparia PPX1^Wt^*, *B. scoparia PPX1^F454I^* (upper two rows of trays) or the untransformed control (lowest row of trays) after spray treatment with the formulation control or carfentrazone-ethyl (0.5x: 8.75 g a.i./ha, 1x: 17.5 a.i./ha or 2x: 35 a.i./ha field use rates) 15 DDA. **(C)** Images are shown of *Arabidopsis* lines ectopically expressing *B. scoparia PPX1^Wt^*, *B. scoparia PPX1^F454I^* (upper two rows of trays) or the untransformed control (lowest row of trays) after spray treatment with the formulation control or fomesafen (0.5x: 105 g a.i./ha, 1x: 210 a.i./ha or 2x: 420 a.i./ha field use rates) 15 DDA.

To analyze whether amino acid substitutions at position F454 in PPO1 are sufficient to confer tolerance to saflufenacil, fomesafen or carfentrazone-ethyl *in planta*, we ectopically expressed the *B. scoparia PPX1* WT or *PPX1* variants encoding F454 substitutions to leucin, isoleucine or valine in the model organism *Arabidopsis thaliana* and assessed the sensitivity of the transgenics to PPO inhibitors. Transgenic lines were treated with the 2x, 1x and 0.5x field dose rates, respectively. Three to five independent T1 transgenic lines expressing the different *PPX1* variants were tested. Consistent herbicide responses were observed across the independent transformants, indicating that the phenotype was reproducible and not restricted to a single transformation event. Based on the visual assessment, ectopic expression of WT *B. scoparia PPX1* did not increase tolerance to saflufenacil and fomesafen at all tested rates. (**Figure 3**; **Supplementary Figure 1**). For carfentrazone-ethyl, WT *B. scoparia PPX1* expression led to a slight reduction in sensitivity at 0.5X field rate which was observed in only one transgenic line but did not confer tolerance at higher rates (**Figure 3**; **Supplementary Figure 1**). In contrast, for saflufenacil and carfentrazone-ethyl two or more independent lines expressing *PPX1* variants encoding F454I, F454L or F454V showed a dramatic increase of tolerance. Notably, saflufenacil tolerance was observed for F454I and F454V substitutions at all rates, whereas no tolerant transgenic lines were observed for the F454L substitution at the 2x rates. For carfentrazone-ethyl, lines expressing *PPX1* variants encoding F454I, F454L or F454V also conferred tolerance at all rates tested (**Figure 3**; **Supplementary Figure 1**). Conversely, at all tested rates for fomesafen, expression of the *PPX1* variants encoding F454 substitutions in transgenic lines did not confer tolerance to fomesafen at all rates tested (**Figure 3**; **Supplementary Figure 1**). Taken together, these findings support that PPO1 variants with F454 substitutions endow resistance to PPO-inhibiting herbicides saflufenacil and carfentrazone-ethyl, while fomesafen efficacy was unaffected.

### 3.6 Molecular modeling of PPO-inhibiting herbicides in *B. scoparia* PPO1 mutant enzymes

Fomesafen, carfentrazone and saflufenacil exhibited different PPO1 inhibitory efficacy in the presence of target-site substitutions at position F454, with fomesafen retaining activity against the F454V, F454L, and F454I variants, while carfentrazone and saflufenacil lose inhibitory potency.

To understand the molecular basis of this differential efficacy, we constructed a homology model of *B. scoparia* PPO1 based on an inhouse co-crystal structure of *Eleusine indica* PPO1 with an inhibitor. The *B. scoparia* PPO1 showed 69% sequence identity and 82% similarity (BLOSUM62) with the *Eleusine* template. Notably, the only residue in direct contact with the herbicide binding site that differs between the two species is phenylalanine (F454 in *B. scoparia*) corresponding to tyrosine (Y418) in *Eleusine*. This model enabled a comparative docking analysis of herbicide binding to both WT and mutant PPO1 variants.

**Figure 4:**
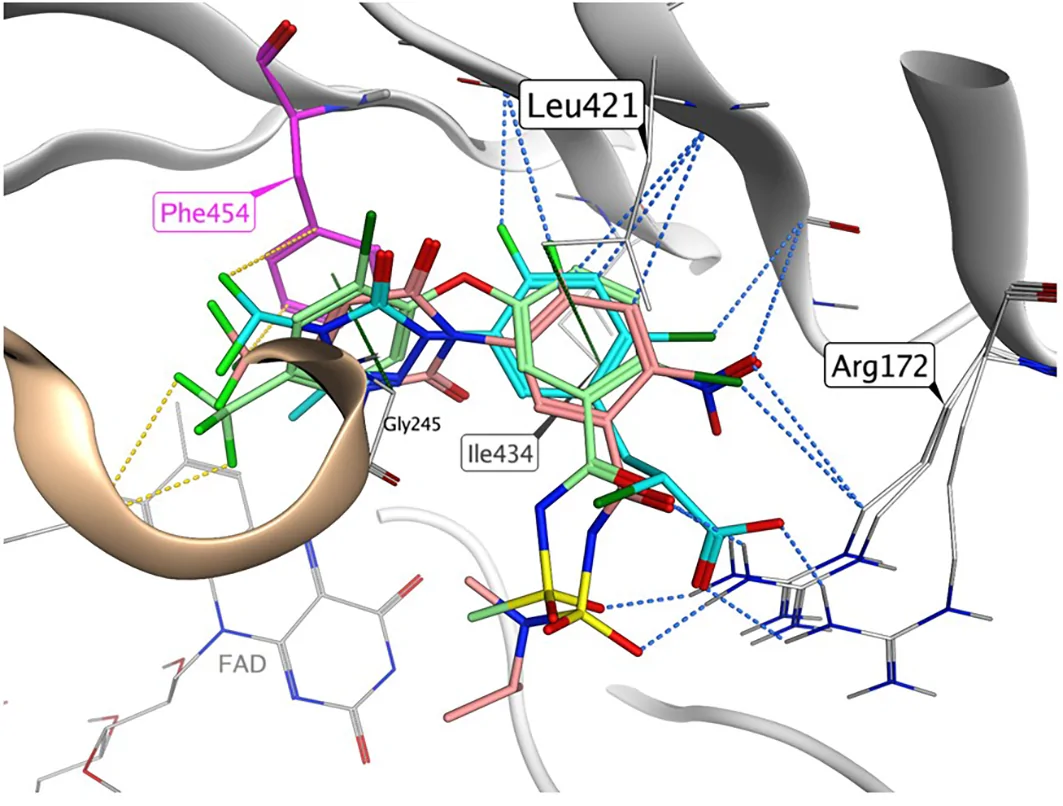
Common ligand interactions of PPO herbicides in the *B. scoparia* PPO1 homology model. The secondary structure of the protein is illustrated in a white cartoon format. The FAD cofactor is represented with grey lines. Oxygen atoms are marked in red, nitrogen atoms in blue, sulfur atoms in yellow, chlorine atoms in dark green, and fluorine atoms in light green. Within the binding pocket, the three herbicides are displayed in stick form: Fomesafen (green carbon atoms), Carfentrazone (cyan carbon atoms), and Saflufenacil (red carbon atoms) are overlaid. The warheads are positioned between Phe454 (magenta carbon atoms) and Gly245 from the alpha helix (gold), allowing for interactions with these amino acids through pi-pi and sigma-pi interactions (green dashed lines). The fluorine group substituents can engage in favorable van der Waals interactions (yellow dashed lines) in this section of the pocket. The scaffolds are situated between Ile434 and Leu421 and are stabilized by sigma-pi interactions (green dashed lines). Additionally, the scaffolds and side chains can interact with the beta sheets of PPO1 through multipolar interactions or charge-assisted hydrogen bonds (blue dashed lines) with Arg172. Arg172 is depicted in different orientations due to its flexible side chain, which can adjust to accommodate the respective herbicide.

Docking simulations were performed using the *B. scoparia* PPO1 homology model, incorporating structural data from multiple PPO inhibitor complexes, including *Nicotiana tabacum* PPO2 (PDB: 1SEZ), ELEIN PPO1, and PPO2 structures co-crystallized with saflufenacil from *A. tuberculatus*. All three herbicides’ binding modes align with typical PPO inhibitor characteristics, with their aromatic warheads—trifluoromethylphenyl (fomesafen), uracil (saflufenacil), and triazole (carfentrazone)—positioned between helix P241–A246 and residue F454, forming canonical π–π interactions (**Figure 4**).

Fomesafen binds to F454 at an angle about 18 degrees different from carfentrazone and saflufenacil, which does not impact the π–π interactions, as F454 can adjust to this variation. However, it results in different exit paths for the trifluoromethylphenyl groups (and CF2 for carfentrazone, which leads to notable hydrophobic interactions to the binding pocket. These interactions to F454 are more pronounced for carfentrazone- and saflufenacil.

**Figure 5:**
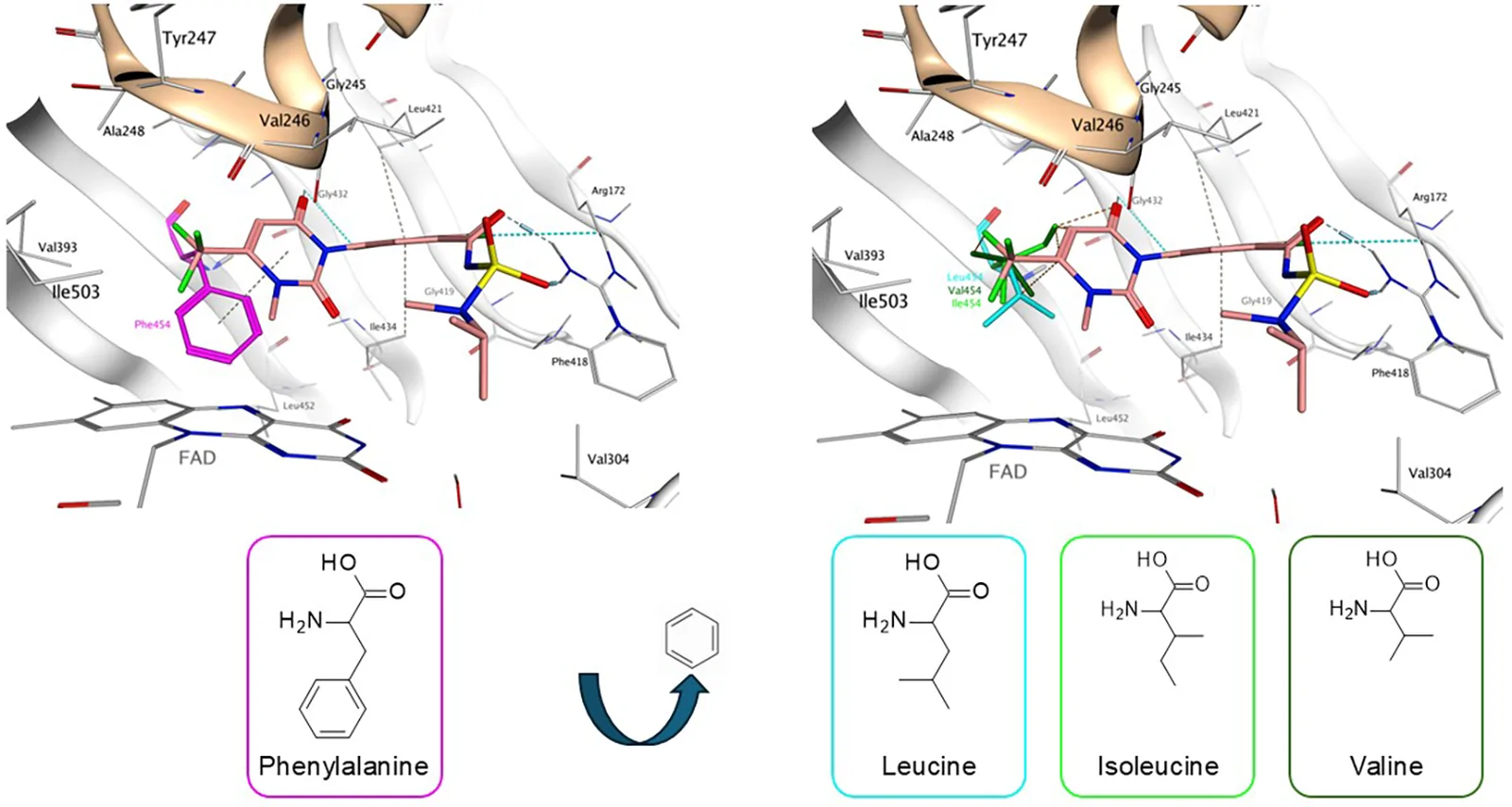
Saflufenacil in the *B. scoparia* PPO1 homology model. The secondary structure of the protein is represented in a white cartoon format. The FAD cofactor is depicted with grey lines. Oxygen atoms are shown in red, nitrogen in blue, sulphur in yellow, chlorine in dark green, and fluorine in light green. Saflufenacil is illustrated in stick form with red carbon atoms. On the left: the uracil warhead of Saflufenacil is positioned between Phe454 (magenta carbon atoms) and Gly245 from the alpha helix (gold), promoting a favorable stacking pi-pi interaction (green dashed line) between Phe454 and the warhead. On the right: substituting Phe454 with Valine (dark green), Isoleucine (green), or Leucine (cyan) eliminates this stacking pi-pi interaction with the phenyl ring side chain of Phe454, and the larger side chains hinder closer binding to the binding site.

Across all herbicides, the molecular scaffolds were twisted by ~60° relative to their warheads, positioning the second ring systems between residues L421 and I434, supporting σ–π interactions (**Figure 4**). The scaffolds can also form multipolar interactions with halogens or polarized hydrogen bonds towards beta-sheet secondary structures. In particular, the nitro group of fomesafen fits well in the binding pocket near the side chain of R172. Furthermore, fomesafen diphenyl ether scaffold includes an additional rotatable bond, providing increased conformational flexibility compared to the rigid heterocyclic frameworks of carfentrazone and saflufenacil. The side chains are exposed to the solvent and can interact with R172 through charge-supported hydrogen bonds. Comparing the three herbicides, differences can be identified between the binding mode of fomesafen to carfentrazone and saflufenacil in PPO1. The F454V, F454L, and F454I substitutions have profound impacts on herbicide binding, primarily by disrupting two critical interaction aspects. First, these mutations abolish π–π stacking interactions that are especially important for carfentrazone and saflufenacil, both of which possess electron-deficient warheads well-suited to such interactions (**Figure 5**). While fomesafen also favors aromatic contacts, its electron-rich phenyl ring allows for σ–π interactions with aliphatic side chains introduced by the mutations. Second, the substitutions introduce steric alterations to the binding pocket. The branched, hydrophobic side chains of valine, leucine, and isoleucine reshape the warhead binding site, affecting additionally the placement of the scaffold. This spatial rearrangement weakens interactions between the scaffold and the β-sheet backbone, which are important for stabilizing inhibitor binding. Fomesafen, with its more flexible ether linkage, can better accommodate these changes by adopting alternative conformations keeping favorable shape complementarity within the altered pocket. In contrast, the rigidity of carfentrazone and saflufenacil limits their ability to adapt to the new binding site geometry.

Taken together, these findings highlight the structural basis for fomesafen retained efficacy against PPO1 variants harboring F454 substitutions. Its increased conformational flexibility, σ–π interaction profile, and ability to adjust to altered pocket geometry render it less prone to resistance than carfentrazoneand saflufenacil. This structural adaptability is a key feature for maintaining herbicidal activity in the presence of target-site substitutions.

### 3.7 Complementation assays with plant PPX genes in a *hem14* yeast mutant

A PPO herbicide screening system in yeast was developed based on conditional lethality using a *hem14/ppx* knockout. This strain can still grow on glucose as carbon source but not when the non-fermentable carbon sources ethanol and glycerol are used in YPGE medium. However, growth on this medium can be restored by complementation with the *B. scoparia PPX1* wildtype and *PPX1* genes encoding F454V I and L substitutions to generate a PPO-inhibitor screening system. When we transformed the *hem14/ppx* knockout with an empty yeast expression vector (p416ADH, Mumberg et al., 1995), the resulting yeast strain can grow at YPD but not on YPGE (**Supplementary Figure 3**). When the yeast was complemented with wildtype *BsPPX1* gene, a differential response of the yeast strains when grown on pH3 YPGE plates compared to pH6 was observed (**Supplementary Figure 4**). Saflufenacil at 200 µM gave higher sensitivity of the yeast expressing wildtype *BsPPX1* at pH3 than at pH6. Conversely, carfentrazone 100 µM gave higher sensitivity of yeast expressing wildtype *BsPPX1* at pH6 than at pH3.

To increase yeast sensitivity to saflufenacil, 500 µM of saflufenacil at pH3 was tested, while carfentrazone 100 µM at pH 6 was tested.

**Figure 6:**
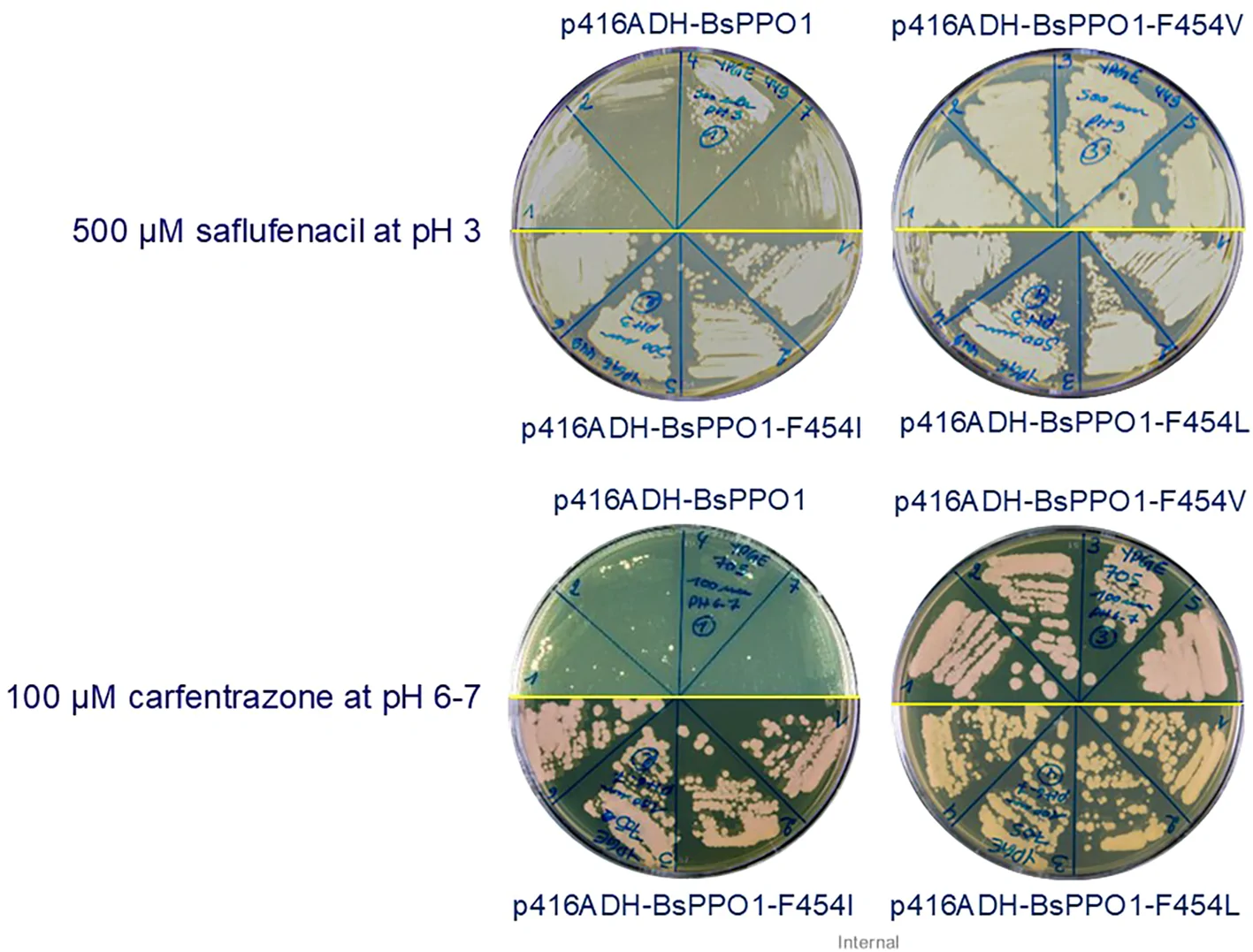
Analysis of p416ADH-BsPPO1 wild-type and mutant variants in yeast strains. Four *B. scoparia BsPPX1* constructs, wild type and encoding substitutions F454I, F454V and F454L, were screened, each with four biological replicates (upper or lower half of each Petri plate) on 500 µM Saflufenacil (pH 3) and 100µm Carfentrazone (pH6-7), respectively.

Severe growth inhibition was observed when WT *B. scoparia PPX1* was expressed in yeast and plated on medium supplemented with 500 µM saflufenacil at pH 3 (**Figure 6**). The four sectors (four biological replicates) remained almost entirely clear, without punctate colonies, indicating strong inhibition of BsPPO1 and loss of yeast viability under herbicide selection. In contrast, yeast strains transformed with *B. scoparia PPX1* constructs encoding the F454V, F454L, or F454I substitutions displayed vigorous growth under the same conditions with the yeast expressing F454V yielding the maximum growth (**Figure 6**). All three variants produced dense, confluent layers of colonies across their inoculated sectors. This strong growth response indicates that each of the F454 substitutions substantially reduces the ability of saflufenacil to inhibit the *B. scoparia* PPO1 enzyme variants in yeast.

When carfentrazone was tested at pH 6–7, yeast expressing the *B. scoparia PPX1* WT again showed no detectable colony formation, with completely clear sectors (**Figure 6**). In contrast, complementation with *PPX1* constructs encoding the F454V, F454L, or F454I substitutions induced abundant yeast growth, with sectors fully covered by large, cream-colored colonies, and yeast expressing *PPX1* encoding the F454V substitution yielded the maximum growth as observed for saflufenacil. The consistent growth advantage of all three mutant variants across two chemically distinct PPO inhibitors demonstrates that substitutions at residue F454 confer cross-resistance to both saflufenacil and carfentrazone. Thus, F454 substitutions are sufficient to disrupt effective herbicide binding while retaining enzyme functionality in the heterologous yeast expression system. When fomesafen was tested, no significant growth inhibition was observed at 500 µM in any of the yeast complemented strains tested, independently of the medium pH (**Supplementary Figures 2A, B**). This suggests that fomesafen is not efficiently taken up or metabolized by the yeast in the tested conditions. Yeast ABC transporters, including PDR5 and YOR1, are known to export a broad range of xenobiotics, including herbicides and fungicides (Rogers et al., 2001). Therefore, their potential contribution to the limited activity of fomesafen in the yeast complementation assay cannot be excluded.

### 3.8 Expression pattern of *PPX1* and *PPX2* during *A. palmeri* and *B. scoparia* early development

**Figure 7:**
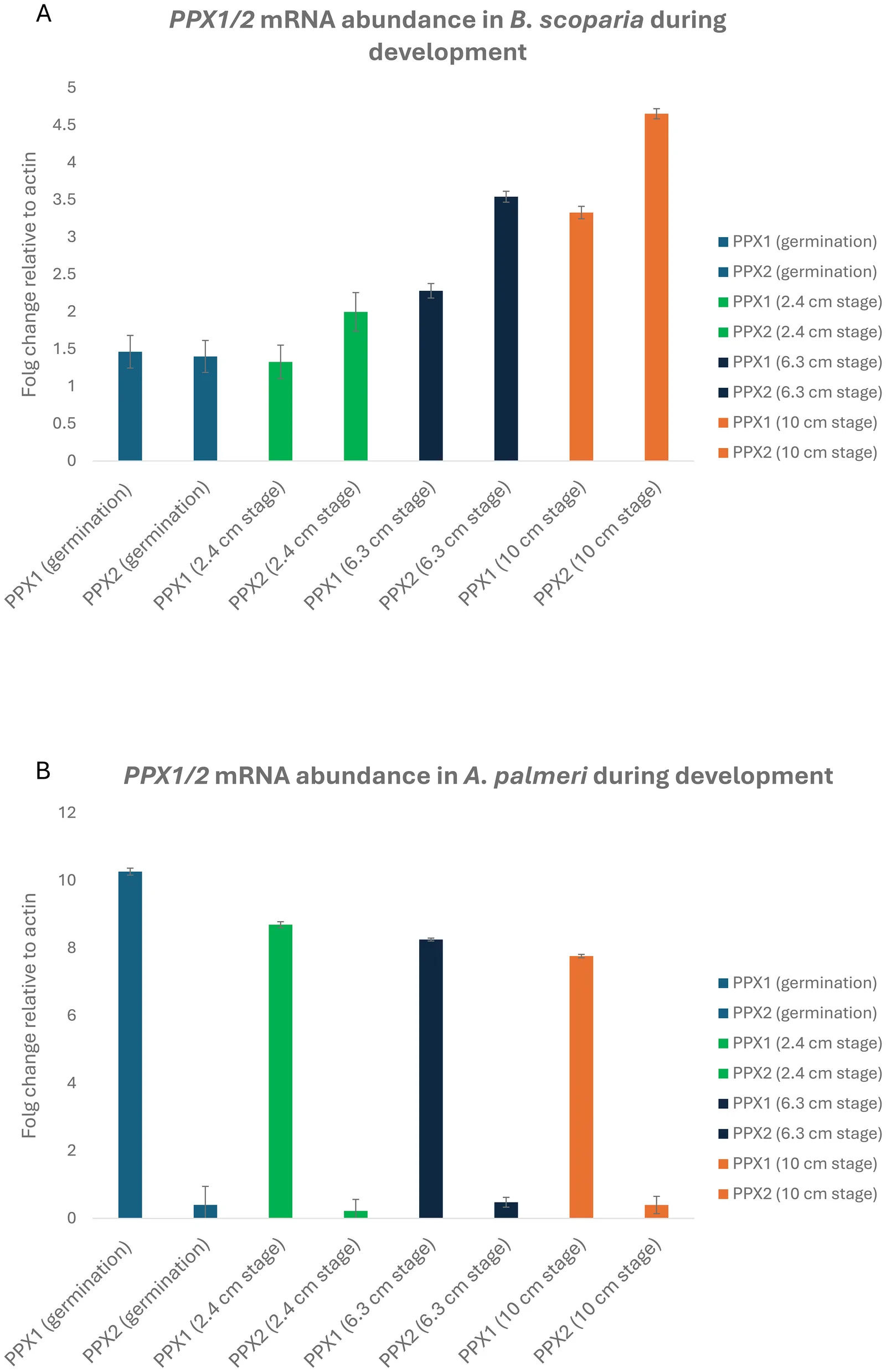
*PPX1* and *PPX2* transcript levels during early seedling development in *B. scoparia* and *Amaranthus palmeri*. Quantitative PCR analysis of *PPX1* and *PPX2* mRNA abundance at four developmental stages in *B. scoparia* **(A)** and *A. palmeri* **(B)** germination, 2.4 cm shoot length, 6.3 cm shoot length, and 10 cm shoot length using digital PCR. Expression levels are presented as fold change relative to *actin*. Bars represent 95% confidence intervals (CI95) of three independent biological replicates.

To investigate the expression dynamics of *PPX1* and *PPX2* during early development of *B. scoparia*, transcript levels were quantified at germination, and three shoot growth stages, 2.4 cm, 6.3 cm, and 10 cm. Expression levels were normalized to *actin* and are presented as fold changes (**Figure 7A**). At the germination stage, both *PPX1* and *PPX2* exhibited moderate expression, with fold-change values of approximately 1.45 and 1.40, respectively. This suggests that both genes are transcriptionally active during early seedling emergence, supporting initial plastid development and tetrapyrrole biosynthesis. At the 2.4 cm stage, *PPX1* expression remained relatively stable at ~1.35-fold, while *PPX2* showed a slight increase to approximately 2.0-fold. Notably, from this point forward, both genes demonstrated a marked and progressive increase in expression levels, coinciding with active shoot elongation. By the 6.3 cm stage, *PPX1* expression rose to ~2.3-fold and *PPX2* to ~3.5-fold. This trend continued into the 10 cm stage, where *PPX1* reached ~3.3-fold and *PPX2* peaked at 4.6-fold, representing over a threefold increase relative to germination. Throughout development, *PPX2* consistently exhibited slightly higher expression levels than *PPX1* from the 2.4 cm stage onward, indicating a potentially more prominent role in later stages of seedling growth. The progressive upregulation of both genes suggests a developmentally regulated demand for porphyrin ring biosynthesis, likely associated with chlorophyll and heme production as the photosynthetic machinery develops.

In addition, expression dynamics of *PPX1* and *PPX2* during early shoot development was also tested in *A. palmeri* at four developmental stages: germination, 2.4 cm, 6.3 cm, and 10 cm shoot length (**Figure 7B**). At the germination stage, *PPX1* showed very high transcript abundance, with a fold change of approximately 10.5, suggesting a strong transcriptional requirement for *PPX1*. In contrast, *PPX2* expression was much lower, below 0.5-fold, suggesting that the PPO2 isoform might play a minor role during seedling emergence. As seedlings elongated to 2.4 cm, *PPX1* expression decreased moderately but remained high (~8.7-fold), while *PPX2* remained very low (~0.3-fold), with no significant induction. This pattern continued into the 6.3 cm and 10 cm stages, where *PPX1* transcript levels remained highly expressed at around 8.2 and 7.8-fold, respectively. In contrast, *PPX2* stayed consistently low, with expression values not exceeding 0.4-fold at any stage. These results demonstrate a striking dominance of *PPX1* expression across all developmental stages in *A. palmeri*, while *PPX2* remains much lower expressed. These data indicate that PPO1 appears to be the primary active isoform throughout seedling development, likely responsible for maintaining plastidic porphyrin ring biosynthesis needed for chlorophyll production and photosynthetic capacity.

## 4 Discussion

Protoporphyrinogen oxidase–inhibiting herbicides remain an important component of weed management programs; however, resistance to this site of action has expanded rapidly in recent years (Heap, 2026). In dicotyledonous weeds, most confirmed cases of PPO-inhibitor resistance have been associated with target-site mutations in *PPX2*, that result in the ΔG210 deletion and substitutions at nearby residues (Patzoldt et al., 2006; Salas et al., 2016; Barker et al., 2023). In contrast, we identify here a distinct resistance mechanism in *B. scoparia* mediated by amino-acid substitutions at F454 in PPO1, resulting in strong resistance to saflufenacil and carfentrazone-ethyl while leaving fomesafen efficacy largely unaffected.

### 4.1 A PPO1-centered target-site resistance mechanism in *B. scoparia*

The resistant *B. scoparia* populations evaluated in this study exhibited high levels of resistance to saflufenacil and carfentrazone-ethyl but remained susceptible to fomesafen. Such herbicide-specific resistance patterns differ from many previously characterized PPO inhibitor-resistant phenotypes, which often show cross-resistance among chemically diverse PPO-inhibiting herbicides (Salas et al., 2016; Rangani et al., 2019; Barker et al., 2023; Riechers et al., 2024; Heap, 2026). Sequencing revealed no known resistance-conferring substitutions in PPO2, and quantitative analyses detected neither CNV nor constitutive overexpression of *PPX1* or *PPX2*, mechanisms that have been implicated in resistance to other herbicide sites of action (Gaines et al., 2020; Gaines et al., 2010; Jugulam et al., 2014).

Instead, resistance was consistently associated with substitutions at phenylalanine 454 F454 in PPO1, occurring as F454I, F454L, or F454V. The recurrence of mutations at this residue across resistant individuals strongly suggests directional selection acting on a critical functional site. Although PPO1 has historically been considered a less common target of resistance evolution than PPO2 (Barker et al., 2023), PPO1-based target-site resistance has been documented previously, notably in *Eleusine indica*, where an A212T substitution in chloroplastic PPO1 confers resistance to oxadiazon (Bi et al., 2020). Our findings report the first ever PPO1 target-site substitutions endowing herbicide resistance in a broadleaf species.

### 4.2 Functional validation confirms a causal role for PPO1 F454 substitutions

A defining feature of this study is the integration of biochemical, genetic, and whole-plant evidence to validate causality. Recombinant PPO1 enzymes carrying F454 substitutions exhibited markedly reduced sensitivity to saflufenacil and carfentrazoneas indicated by elevated IC_50_ values, while sensitivity to fomesafen was largely unchanged. Similar enzyme-level shifts have been used to confirm PPO target-site resistance in *Amaranthus* species and other weeds (Patzoldt et al., 2006; Rangani et al., 2019; Barker et al., 2023; Riechers et al., 2024). Transgenic expression of mutant *PPX1* alleles in *Arabidopsis thaliana* further confirmed that these substitutions are sufficient to confer resistance at the whole-plant level. Plants expressing *PPX1* variants encoding F454I, F454L, or F454V survived rates of saflufenacil and carfentrazone that were lethal to WT controls, while remaining susceptible to fomesafen. These results parallel functional validation approaches used for PPO2 mutations such as ΔG210 and R128 substitutions (Riggins and Tranel, 2012; Salas et al., 2016) and provide strong evidence that resistance in *B. scoparia* arises from altered PPO1 target-site sensitivity rather than non-target-site mechanisms.

### 4.3 Structural basis of herbicide-specific resistance

Structural modeling suggests that F454 occupies a key position within the PPO1 active site, contributing aromatic and hydrophobic interactions that stabilize binding of certain PPO inhibitors. Replacement of phenylalanine with aliphatic residues is predicted to weaken these interactions, thereby reducing binding affinity for saflufenacil and carfentrazone In contrast, fomesafen appears to rely less heavily on this aromatic contact, consistent with its retained efficacy against F454-substituted PPO1 variants.

These findings align with prior structural and biochemical analyses demonstrating that PPO inhibitors differ substantially in their binding modes and interaction networks within the active site (Koch et al., 2004; Dayan and Duke, 2014). Herbicide-specific resistance resulting from subtle alterations in active-site architecture has also been reported for other PPO mutations and underscores why single amino-acid substitutions do not always produce uniform cross-resistance across the PPO inhibitor classes (Dayan et al., 2018).

### 4.4 Implications for resistance evolution and weed management

The identification of PPO1-mediated resistance in *B. scoparia* expands the known spectrum of target-site mechanisms conferring resistance to PPO-inhibiting herbicides. Unlike the *PPX2* mutations previously reported in broadleaf weeds (Patzoldt et al., 2006; Salas et al., 2016; Barker et al., 2023), the F454 protein substitutions identified here confer herbicide-specific resistance, with fomesafen retaining substantial activity. This observation suggests that not all PPO inhibitors are equally affected by PPO1 substitutions and may provide additional management options where fomesafen is registered and agronomically appropriate. However, reliance on a single effective PPO inhibitor would likely increase selection pressure for additional resistance mechanisms, including alternative target-site mutations or non-target-site resistance, both of which are well documented in PPO inhibitor-resistant weeds (Dayan et al., 2018; Rangani et al., 2019; Riechers et al., 2024).

Our findings also demonstrate that PPO1 represents an underrecognized evolutionary target for herbicide resistance. To date, resistance monitoring has focused primarily on *PPX2* because all validated target-site mutations in broadleaf weeds have been identified in this gene (Barker et al., 2023). The discovery of PPO1 F454 substitutions indicates that future resistance diagnostics should include both PPO isoforms to avoid overlooking emerging resistance mechanisms.

*B. scoparia* has evolved resistance to multiple herbicide sites of action and remains one of the most problematic weeds across the North American Great Plains (Kumar et al., 2019; Geddes et al., 2025a). Consequently, the continued effectiveness of available herbicides will depend on their integration with diversified weed management practices that reduce selection pressure and limit seed production (Osipitan et al., 2019; Mosqueda et al., 2020; Obour et al., 2021; Tidemann et al., 2017; Tidemann et al., 2024; Dhanda and Kumar, 2025; Geddes and Davis, 2021; Beckie et al., 2016). Although the herbicide-specific resistance identified here suggests that certain PPO chemistries may retain efficacy, sustainable management will require integrated strategies alongside continued investment in the discovery of new herbicide chemistries.

Beyond the immediate implications for resistance diagnostics, our findings suggest that future resistance monitoring programs should routinely evaluate both *PPX1* and *PPX2* to capture the full diversity of emerging PPO target-site resistance mechanisms. As resistance to PPO inhibitors continues to evolve globally, integrating molecular diagnostics with coordinated resistance surveillance networks will be essential for the early detection and tracking of novel resistance alleles. Furthermore, the structural insights obtained here highlight how subtle changes within the PPO1 binding pocket differentially affect inhibitor binding. These findings provide a valuable framework for the rational design of next-generation PPO inhibitors with improved resilience against evolving target-site resistance. Although the greenhouse experiments were evaluated using standardized visual control ratings rather than quantitative biomass measurements, the objective was to compare the herbicide-specific responses of PPO1 mutant populations across commercially relevant application rates. Importantly, the resistance status and resistance indices of the Mandan and Minot populations had already been established through conventional dose-response analyses in Geddes et al. (2025). Consequently, the present study extends those findings by defining the herbicide-specific response profile associated with the newly identified PPO1 F454 substitutions. Future studies incorporating quantitative biomass measurements will enable complementary GR_50_ estimates for newly identified PPO1 mutant populations.

## Data Availability

The nucleotide sequence data generated in this study have been deposited in the NCBI GenBank database under accession number BankIt ID 3083452.
