# Correction: Novel amino acid substitutions in Protoporphyrinogen oxidase 1 endow resistance to PPO-inhibiting herbicides in *Bassia scoparia*

**DOI:** 10.3389/fpls.2026.2019172

**Published:** 2026-10-22

**Authors:** Frontiers Production Office

**Affiliations:** Frontiers Media SA, Lausanne, Switzerland

**Keywords:** *B. scoparia*, herbicide resistance, PPO, PPO-inhibiting herbicides, weeds

The **Copyright** statement for this article was incorrectly written as “Copyright © 2026 Porri, Law, Geddes, Ikley, Willingham, Johnen, Meiners, Al-Sammarraie, Crommar, Ouwerkerk, Betz, Jenks, Heiser, Baumann, Braendle and Lerchl.” Copyright in respect of Charles M. Geddes’ portion of the article belongs to His Majesty the King in Right of Canada. The correct copyright statement is:

“Copyright © 2026 Porri, Law, Ikley, Willingham, Johnen, Meiners, Al-Sammarraie, Crommar, Ouwerkerk, Betz, Jenks, Heiser, Baumann, Braendle, and Lerchl and His Majesty the King in Right of Canada, as represented by the Minister of Agriculture and Agri-Food Canada for the contribution of Geddes. This is an open-access article distributed under the terms of the Creative Commons Attribution License (CC BY). The use, distribution or reproduction in other forums is permitted, provided the original author(s) and the copyright owner(s) are credited and that the original publication in this journal is cited, in accordance with accepted academic practice. No use, distribution or reproduction is permitted which does not comply with these terms.”

In the original article, Affiliation 3 was incorrectly written as “Gouvernement du Canada Agriculture et Agroalimentaire Canada, Ottawa, Canada”. This should be:

“Agriculture and Agri-Food Canada, Lethbridge, AB, Canada”

The original version of this article has been updated.

