# Supplementary material for: Novel amino acid substitutions in Protoporphyrinogen oxidase 1 endow resistance to PPO-inhibiting herbicides in Bassia scoparia
Source: Front Plant Sci. 2026 Sep 11;17:1904705. doi: 10.3389/fpls.2026.1904705 (PMC13612512; doi:10.3389/fpls.2026.1904705)
Supplement: Supplementary file 1 [file Table1.docx]

**Supplementary figures and tables**

**Table S1.** Others PPO2 aa substitutions detected in ***B. scoparia*** plants surviving 1x Carfentrazone-ethyl (A) and 1x Saflufenacil treatments (B), identified by Sanger sequencing. The / indicates that the one allele carries the wild-type amino acid while the other the amino acid substitution.

| **A** |  |  | |
| --- | --- | --- | --- |
| Biotype | B. scoparia PPO2 | I201 | V331 |
|  | Amaranth PPO2 | V235 | V365 |
| **Berthold** | Survivor ID  Survivor 1 | I | V |
|  | Survivor 2 | I | V |
|  | Survivor 3 | I/L | V |
| **Mandan** | Survivor 1 | I | V |
|  | Survivor 2 | I | V |
|  | Survivor 3 | I/L | V |
|  | Survivor 4 | I/L | V |
| **Minot** | Survivor 1 | I | V/F |
|  | Survivor 2 | I | V |
|  | Survivor 3 | I | V |
| **Mott** | Survivor 1 | L | V/F |
|  | Survivor 2 | L | V/F |
|  | Survivor 3 | I | V/F |
| **B** |  |  |  |
| **Berthold** | Survivor 1 | I | V |
|  | Survivor 2 | I | V/F |
|  | Survivor 3 | I | V |
| **Mandan** | Survivor 1 | I | V |
|  | Survivor 2 | I | V |
|  | Survivor 3 | I | V |
|  | Survivor 4 | I/L | V |
| **Minot** | Survivor 1 | I/L | V |
|  | Survivor 2 | I | V |
|  | Survivor 3 | I | V/F |
| **Mott** | Survivor 1 | I | V |
|  | Survivor 2 | I | V |

**Table. S2. Sequences of the primers and probes employed in the dPCR to determine CNV and expression levels of *PPX1* and *PPX2* in *B. scoparia* survivor plants to 1x Saflufenacil and 1x Carfentrazone-ethyl.**

| Name | Sequence | Fluorophore |
| --- | --- | --- |
| PPX1-dPCR-for | GCCTAAGCCGCCTCGAGATCCG |  |
| PPX1-dPCR-rev | GCCTAGCAGAAATGGCGTTCGGC |  |
| PPX1-dPCR-probe | CGCCTTCCGAAGCCAAAGGGTCAGACAGT | Fam / BMN-Q535 |
| PPX2-dPCR-for | TGAGAAGCTATTGGGCACTGAGGGT |  |
| PPX2-dPCR-rev | TGCTCGATTGCTTTGAGTACTGAGTCA |  |
| PPX2-dPCR-probe | GGAGCAAGGCATTCCCTCTTTATGGGCGCA | Hex/BMN-Q535 |
| Actin-dPCR-for | AACAAGGTCAACGATTGCGG |  |
| Actin-dPCR-rev | CTCCAGCCGCCTCTTTAGAG |  |
| Actin-dPCR-probe | GCTGAGGCAAAGAGGGCAGCTCGG | ROX / BMN-Q620 |

**Table S3** Overview of yeast strains with or without *PPX* complementation constructs used in this study.

| **Strain** | **Genotype** | ***PPX* gene** |
| --- | --- | --- |
| YSA0024 | MATα his3Δ1 leu2Δ0 ura3Δ0 lys2Δ0 hem14::kanMX4 p416ADH-BsPPO1 | *BsPXO1* |
| YSA0025 | MATα his3Δ1 leu2Δ0 ura3Δ0 lys2Δ0 hem14::kanMX4 p416ADH-BsPPO1 F454I | *BsPXO1 F454I* |
| YSA0026 | MATα his3Δ1 leu2Δ0 ura3Δ0 lys2Δ0 hem14::kanMX4 p416ADH-BsPPO1 F454V | *BsPPX1 F454V* |
| YSA0027 | MATα his3Δ1 leu2Δ0 ura3Δ0 lys2Δ0 hem14::kanMX4 p416ADH-BsPPO1 F454L | *BsPPX1 F454L* |

**Fig. S1.** *Arabidopsis* lines in the T1 Generation ectopically expressing indicated ***B. scoparia*** *PPX1* variants were sprayed with saflufenacil, carfentrazone-ethyl or fomesafen. The upper three to five pots contain independent transgenic events (T1 plants, were selected with Imazamox to confirm the presence of resistance gene T-DNA via AHAS herbicide tolerance) the two plants in the lowest row represent the untransformed MC24 wild type control. (A) Images are shown of *Arabidopsis* lines ectopically expressing *B. scoparia PPX^Wt^* (same control as presented in Fig. XY), *B. scoparia PPX^F45LI^*, *B. scoparia PPX^F454V^* (upper three rows of trays) or the untransformed control (lowest row of trays) after spray treatment with the formulation control or saflufenacil (0.5x: 12.5 g a.i./ha, 1x: 25 a.i./ha or 2x: 50 a.i./ha field use rates) 11 days after treatment. (B) Images are shown of *Arabidopsis* lines ectopically expressing *B. scoparia PPX^Wt^* (same control as presented in Fig. XY), *B. scoparia PPX^F45LI^*, *B. scoparia PPX^F454V^* (upper two rows of trays) or the untransformed control (lowest row of trays) after spray treatment with the formulation control or carfentrazone-ethyl (0.5x: 8.75 g a.i./ha, 1x: 17.5 a.i./ha or 2x: 35 a.i./ha field use rates) 15 days after treatment. (C) Images are shown of *Arabidopsis* lines ectopically expressing *B. scoparia PPX^Wt^* (same control as presented in Fig. XY), *B. scoparia PPX^F45LI^*, *B. scoparia PPX^F454V^* (upper two rows of trays) or the untransformed control (lowest row of trays) after spray treatment with the formulation control or fomesafen 0.5x: 105 g a.i./ha, 1x: 210 a.i./ha or 2x: 420 a.i./ha field use rates) 15 days after treatment.

**Fig. S2. A and B. Herbicide screening assays to check for tolerance levels for fomesafen at 500 µM.** Fomesafen was screened at 500 µM but because of solubility issues, this herbicide was spread at the medium plates before inoculation of the yeast strains. As a HEM14 wild type control, BY4741 was used which is the MATa parent of BY4743 (Baker Brachmann et al., 1998) from which YKC027 originates. YKC029 and YKC033 contain *A. palmeri* *PPX2* and *PPX1* constructs, respectively, complementing the hem14::kanMX4 allele that were used as control strains too. Four *B. scoparia BsPPX1* constructs, econdign the variants F454I, F454V and F454L, were screened, each with four replicates (upper half of each Petri plate).

**Figure S3. Empty-vector control confirms the requirement for PPX1-mediated complementation in the yeast assay.** The *Saccharomyces cerevisiae* hem14 knockout strain (YKO27) transformed with the empty expression vector (p416ADH) was grown on glucose-containing medium (left) or on non-fermentable YPGE medium containing glycerol and ethanol (right). Yeast carrying the empty vector grew normally on glucose medium but failed to grow on YPGE medium, demonstrating that the empty vector does not complement the *hem14* knockout. These results confirm that growth on YPGE requires expression of a functional plant PPX1 gene and validate the complementation assay used to evaluate PPO inhibitor sensitivity.

**Figure S4. Analysis of p416ADH-BsPPO1 wild-type and mutant strains on (A) Saflufenacil and (B) Carfentrazone.** Four *B. scoparia* *BsPPO1* constructs, wild type and encoding substitutions F454I, F454V and F454L, were screened, each with four biological replicates (upper half of each Petri plate) on 1, 10 or 100 µM Saflufenacil or Carfentrazone (pH 3 or 6-7), respectively. On the lower halves we had control strains BY4741 (wild type HEM14), YKC029 (AmPPO2 replacing HEM14) and YKC033 (AmPPO1 replacing HEM14).

**References**

**Baker Brachmann, C., Davies, A., Cost, G. J., Caputo, E., Li, J., Hieter, P., & Boeke, J. D. (1998).** *Designer deletion strains derived from Saccharomyces cerevisiae S288C: A useful set of strains and plasmids for PCR-mediated gene disruption and other applications.* **Yeast, 14**(2), 115–132. https://doi.org/10.1002/(SICI)1097-0061(19980130)14:2
